# Nerve Guidance Conduit Prepared from Decellularized Small Intestine for Nerve Repair

**DOI:** 10.3390/jfb17040170

**Published:** 2026-04-01

**Authors:** Xiang-Ting Huang, Ying-Chih Lin, Ling-Yun Cheng, Yi-Dan Chang, Wen-Yu Su

**Affiliations:** 1 Department of Bioinformatics and Medical Engineering, Asia University, Taichung 41354, Taiwan; 2 Horien Biochemical Technology Co., Ltd., Taichung 40768, Taiwan

**Keywords:** peripheral nerve regeneration, nerve guidance conduit, small intestinal submucosa, decellularized extracellular matrix, biodegradable braided scaffold

## Abstract

Braided nerve guidance conduits (NGCs) composed of decellularized porcine small intestinal submucosa (SIS) were developed to achieve an appropriate balance between mechanical performance and biological compatibility for peripheral nerve repair. This study aimed to compare four SIS-braided conduits with silicone tubes in terms of bending compliance, tensile strength, swelling behavior, and cytocompatibility. SIS-braided conduit exhibited a favorable combination of flexibility, tensile strength, and dimensional stability. In vitro evaluations using PC12 and SW10 cells demonstrated that SIS-braided conduit supported neurite outgrowth and Schwann cell adhesion, confirming its favorable cytocompatibility. Based on these findings, SIS-braided conduits and silicone tubes were subsequently evaluated in a rat sciatic nerve defect model. Functional recovery assessed using the Sciatic Functional Index suggested preliminary functional recovery in the SIS-braided conduit, and histological analyses revealed evidence of axonal regeneration and myelin formation within the conduit. Overall, the results indicate that the integration of mechanical robustness with biological activity is essential for the design of nerve graft substitutes. The conduit braided from decellularized small intestinal submucosa represents a promising biodegradable alternative, a considerable biodegradable alternative to conventional non-degradable silicone conduits for peripheral nerve repair.

## 1. Introduction

The human nervous system is composed of the central nervous system (CNS) and the peripheral nervous system (PNS). The CNS, consisting of the brain and spinal cord, is responsible for processing information and coordinating the body’s functions and responses. The PNS, comprising nerves outside the brain and spinal cord, transmits signals between the CNS and the rest of the body, enabling communication and control of motor and sensory functions [1]. Peripheral nerve injury is a common clinical problem, with an estimated incidence of 13.9–23 cases per 100,000 individuals annually [2]. As the global population continues to grow and age, the number of affected individuals has increased significantly, especially in low- and middle-income countries [3]. Based on Taiwan’s total population in 2023, the potential patient number is approximately 3247. Such injuries may result from lacerations, crush injuries, or traction-related damage to nerves [4]. In the United States, annual expenditures related to nerve graft surgeries are estimated at around 7 billion USD, with approximately 50,000 procedures performed each year [5].

Treating peripheral nerve injury (PNI) remains a significant clinical challenge. PNI commonly results from trauma, accidents, combat injuries, or certain diseases, and it severely affects patients’ quality of life and functional recovery. In China, approximately one million new cases of PNI occur annually, with 200,000–300,000 cases of nerve defects requiring repair using nerve grafts [6]. With the rapid advancement of science and technology, particularly in tissue engineering, the development of tissue-engineered grafts for peripheral nerve repair has shown great potential [7]. Such grafts are expected to possess excellent biocompatibility and biodegradability, making them attractive alternatives to conventional autologous or synthetic nerve grafts [8].

Nerve conduits have shown promise in repairing peripheral nerve injuries (PNIs), which often result in severe motor and sensory deficits. Although peripheral nerves can regenerate spontaneously, this is usually insufficient, especially in cases of complete transection [9]. Recovery is influenced by factors such as nerve gap length, treatment timing, and patient age. Treatments include non-surgical approaches, such as electrical stimulation, laser therapy, and nerve growth factors, as well as surgical interventions, including direct repair, grafts, and tissue-engineered conduits [10]. Although autologous nerve grafting remains the clinical gold standard for bridging nerve gaps, its application is limited by donor-site morbidity, limited graft availability, and potential size mismatches between donor and recipient nerves. Nerve conduits provide a biocompatible and biodegradable scaffold that supports axonal growth without the need for donor tissue or permanent synthetic implants, offering a promising alternative for functional recovery [11,12]. To address these limitations, nerve guidance conduits have emerged as promising alternatives, providing biocompatible, biodegradable scaffolds that support axonal regeneration without donor nerve harvests or permanent implants. They are increasingly recognized as effective strategies to enhance functional recovery and advance nerve repair approaches [13]. Nerve guidance conduits (NGCs) connect proximal and distal nerve stumps, directing axonal growth while preventing scar formation and protecting regenerating nerves from mechanical stress [14]. While synthetic polymers such as silicone have been used, their non-degradable nature can induce chronic inflammation, prompting growing interest in natural biomaterials [15,16].

Small intestinal submucosa (SIS), a decellularized extracellular matrix (ECM) derived from porcine small intestine, is a particularly promising candidate [17]. Among these materials, small intestinal submucosa (SIS), a decellularized extracellular matrix derived from porcine intestine, has attracted attention due to its favorable biocompatibility, preserved Extracellular Matrix architecture, and potential to support tissue regeneration. SIS retains collagen, glycosaminoglycans (GAGs), and basic Fibroblast Growth Factor (bFGF) that support cell adhesion, migration, and differentiation [18]. In addition, its biodegradability, proangiogenic activity, and immunomodulatory properties make SIS particularly suitable for nerve tissue engineering [19].

Previous studies have shown that SIS-based sheets and tubular scaffolds can effectively support peripheral nerve regeneration in small animal models [20,21,22]. Small intestinal submucosa (SIS) has been widely explored as a natural extracellular matrix scaffold for peripheral nerve repair, and SIS-based conduits have shown promising outcomes in preclinical models and limited clinical applications. However, conventional SIS conduits are typically fabricated as rolled sheets or multilayer tubes, which may present limitations in flexibility and resistance to kinking. Therefore, developing alternative conduit architectures that maintain lumen patency while preserving the bioactivity of SIS remains an important research direction [23,24].

To address the limitations of existing nerve conduits, we developed braided nerve guidance conduits using decellularized small intestinal submucosa (SIS). Braiding SIS fibers into tubular scaffolds preserves the material’s intrinsic bioactivity while enhancing mechanical strength, flexibility, suture retention, and intraoperative handling. This architecture also provides a supportive microenvironment for axonal growth. We evaluated these conduits in repairing critical-sized sciatic nerve defects in a rat model. The braided SIS conduits promoted axonal regeneration and functional recovery, suggesting that integrating SIS biodegradability with the mechanical advantages of braiding represents a promising strategy for peripheral nerve repair and regeneration.

## 2. Materials and Methods

### 2.1. Materials and Reagents

Small intestinal submucosa (SIS) was obtained from Horien Biochemical Technology Co., Ltd. (Taichung, Taiwan). Alkyl polyglucoside (APG; BASF, Ludwigshafen, Germany), porcine pancreatic trypsin (Merck, Darmstadt, Germany), and ethylenediaminetetraacetic acid (EDTA; Merck, Darmstadt, Germany) were used for decellularization. Rat pheochro-mocytoma cells (PC12; BCRC/FIRDI, Hsinchu, Taiwan) and rat Schwann cells (SW10; BCRC/FIRDI, Hsinchu, Taiwan) were used for in vitro evaluation. RPMI 1640 (Gibco, Waltham, MA, USA) and Dulbecco’s Modified Eagle Medium (DMEM; Gibco, Waltham, MA, USA) were purchased from Gibco (Thermo Fisher Scientific, Waltham, MA, USA) and supplemented with 10% fetal bovine serum (FBS; HyClone, Logan, UT, USA) and 1% Antibiotic-Antimycotic (Gibco, Waltham, MA, USA). The WST-1 assay kit and LDH Cytotoxicity Detection Kit were obtained from Roche (Mannheim, Germany).

### 2.2. Preparation of SIS Nerve Guidance Conduit

**Figure 1:**
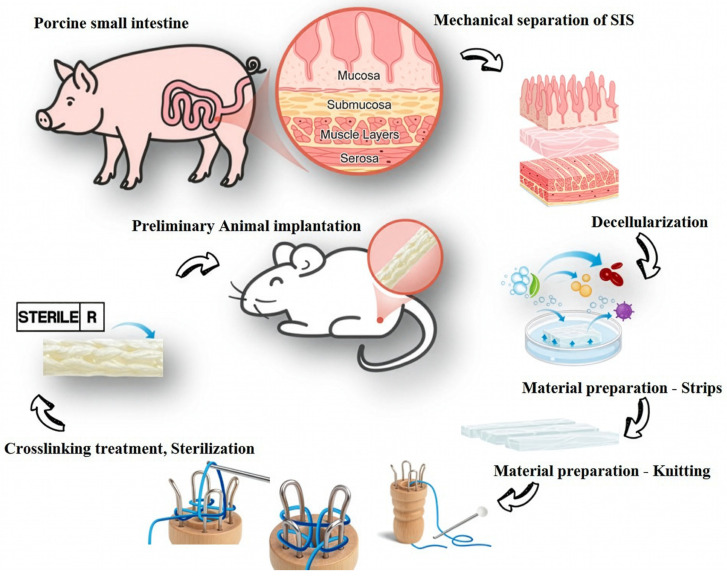
Preparation of SIS nerve conduits. The submucosa layer was mechanically separated from porcine small intestine, decellularized, and cut into strips. The strips were then braided, crosslinked, and sterilized before implantation for 12-week sciatic nerve regeneration.

Porcine small intestines were obtained from a local slaughterhouse. The small intestinal submucosa (SIS) tissue was sourced from Lanyu Yi-Du (LYD) miniature pigs aged 6–8 months, with an average body weight of approximately 58.3 kg (50-head batch). The small intestinal submucosa (SIS) layer was mechanically separated. The isolated SIS was subsequently decellularized using the APG method and thoroughly washed to remove residual detergents and cellular debris [25]. The decellularized SIS sheets were cut into strips measuring 15 mm or 20 mm in width, rolled into tubular forms, and braided using a knitting machine to produce four-needle (CK154, CK156) and six-needle (CK204, CK206) conduit configurations. A 1.98 mm diameter Teflon mandrel was used to maintain lumen patency during fabrication (Figure 1). The fabricated conduits were subjected to dehydrothermal (DHT) crosslinking at 110 °C for 24 h to enhance the structural stability of the extracellular matrix, followed by gamma irradiation (25 kGy) for sterilization prior to in vitro and in vivo experiments.

### 2.3. Surface Chemistry Characterization by Fourier Transform Infrared Spectroscopy

The functional groups of the prepared samples were analyzed using a Fourier-Transform Infrared Spectroscopy (FTIR) spectrometer (Nicolet Summit LITE, Thermo Fisher Scientific) equipped with an attenuated total reflectance (ATR) accessory. Spectra were recorded over the range of 4000–600 cm^−1^ with a spectral resolution of 4 cm^−1^.

### 2.4. Surface Morphology and Decellularization Characterization

The decellularization procedure (APG method) was conducted as previously described [25]. Decellularization was evaluated by Elastic Van Gieson (EVG) staining to assess the presence or absence of cellular components. Residual DNA was analyzed by gel electrophoresis following extraction from native and decellularized SIS samples (PrepSEQ™ Residual DNA Sample Preparation Kit, Thermo Fisher Scientific). Surface morphology was examined using scanning electron microscopy (SEM; TM-1000, Hitachi, Tokyo, Japan). Samples were freeze-dried, mounted on aluminum stubs with carbon tape, and sputter-coated with gold (E-1010, Hitachi, Tokyo, Japan) prior to imaging.

### 2.5. Swelling and Degradation Evaluation

The swelling and degradation behaviors of the nerve guidance conduits (NGCs) were evaluated by measuring their weight changes before and after immersion in physiological saline.

Swelling ratio (%) = (W_1_ − W_0_)/W_0_ × 100 (1)

where W_0_ represents the initial dry weight and W_1_ represents the weight of the conduit after immersion in physiological saline.

For degradation analysis, only the CK154 braided sample was evaluated. CK154 was selected based on its more suitable swelling behavior, suture retention strength, bending flexibility, and kink resistance among the tested conduits. The conduits were collected after the designated incubation period, dried to a constant weight, and weighed to obtain the final dry weight (Q_1_). The degradation ratio was calculated as follows:

Degradation ratio (%) = (Q_1_ − Q_0_)/Q_0_ × 100 (2)

where Q_0_ represents the initial dry weight and Q_1_ represents the final dry weight of the conduit after incubation and drying.

### 2.6. In Vitro Cytotoxicity

PC12 cells (rat pheochromocytoma cell line) and SW10 cells (mouse Schwann cell line) were obtained from Gibco and cultured in Dulbecco’s Modified Eagle Medium (DMEM, high glucose) supplemented with 10% fetal bovine serum (FBS) and 1% penicillin–streptomycin under standard culture conditions (37 °C, 5% CO_2_).

Prior to testing, cells were seeded into 96-well plates at a density of 5 × 10^3^ cells per well and allowed to adhere overnight. Experimental groups included extract solutions from braided SIS conduits and silicone tubes, prepared according to ISO 10993-12 [26], alongside a negative control (culture medium only) and a positive control (0.1% Triton X-100 for LDH lysis).

Cell viability and metabolic activity were assessed using the WST-1 assay (Roche) on Day 1 and Day 3 post-treatment. At each time point, WST-1 reagent (10% of culture volume) was added to each well and incubated for 2 h at 37 °C. Absorbance was measured at 450 nm, and cell viability (%) was calculated relative to the negative control. Cell membrane integrity was evaluated by measuring lactate dehydrogenase (LDH) release using the LDH Cytotoxicity Detection Kit (Roche) on Day 1 and Day 3 post-treatment. At each time point, 50 µL of cell culture supernatant was transferred to a new 96-well plate, mixed with the reaction mixture, and incubated for 30 min at room temperature in the dark. Absorbance was measured at 490 nm using a microplate reader.

Cell viability (%) = (Exp − Blank)/(Control − Blank) × 100(3)

Live/Dead fluorescence imaging was performed using the LIVE/DEAD^®^ Viability/Cytotoxicity Kit (Thermo Fisher Scientific) to assess cell morphology and viability. PC12 and SW10 cells were stained with calcein AM (live, green) and ethidium homodimer-1 (dead, red), and fluorescence images were acquired.

For quantitative analysis, images were processed using ImageJ (version 1.54g) by splitting RGB channels, followed by background subtraction and thresholding (Otsu method). Small artifacts (<20 pixels) were excluded, and live and dead cells were quantified using the Analyze Particles function (size: 20–Infinity). Cell viability (%) was calculated as live/(live + dead) × 100, complementing WST-1 results.

### 2.7. Suture Retention Strength

The suture retention and tensile strength of the nerve guidance conduits were evaluated using a universal testing machine. Both ends of each conduit sample were sutured with 8-0 suture threads, which were carefully wrapped around the material to simulate surgical fixation. Mechanical testing was conducted at a constant crosshead speed of 10 mm/min to assess the maximum force the suture-material interface could withstand, providing a quantitative measure of suture retention and seam integrity [27].

### 2.8. Bending Flexibility and Kink Resistance Test

The bending and kinking behavior of the nerve guidance conduits was assessed by inserting a 1.25 mm diameter stainless steel wire into the lumen to provide structural support. The conduits were sequentially bent to angles of 0°, 30°, 60°, and 90°, and a protractor was used to measure the bending angles accurately. At each angle, the lumen was visually inspected for kinking, collapse, or any structural deformation. This evaluation provided a quantitative assessment of the conduits’ flexibility and resistance to bending-induced lumen obstruction [28].

### 2.9. In Vivo Evaluation in a Rat Sciatic Nerve Defect Model

Adult male Sprague–Dawley rats 7–8 weeks old, weighing 200–250 g, were used and housed under standard laboratory conditions with a 12 h light/dark cycle and free access to food and water. All animal experiments were conducted in accordance with institutional and national guidelines for the care and use of laboratory animals. The experimental protocol was reviewed and approved by the Institutional Animal Care and Use Committee (IACUC) of Comewin Bio Co., Ltd., Taichung, Taiwan (Approval No. IACUC #25005).

Rats were anesthetized via intraperitoneal injection, and a gluteal muscle–separating incision was made to expose the left sciatic nerve, which was transected to create a critical-size defect. The nerve gap was bridged using either a silicone tube (SCT) or a braided small intestinal submucosa (SIS) nerve conduit (CK154), secured to the nerve stumps with 8-0 nylon sutures, and the muscle and skin were subsequently closed in layers. Functional recovery was evaluated using the Sciatic Functional Index (SFI) at 4, 8, and 12 weeks post-surgery. Rats’ hind paws were coated with non-toxic Crayola Washable Spill-Proof Paint and allowed to walk along a confined corridor lined with white paper [29]. Parameters measured included print length (PL), toe spread (TS), and intermediary toe spread (IT) for both the experimental (E) and normal (N) limbs. The SFI was calculated by the following equation

SFI = −38.3 × (EPL − NPL)/NPL + 109.5 × (ETS − NTS)/NTS + 13.3 × (EIT − NIT)/NIT − 8.8(4)

where EPL = experimental print length, NPL = normal print length, ETS = experimental toe spread, NTS = normal toe spread, EIT = experimental intermediary toe spread, and NIT = normal intermediary toe spread.

An SFI of 0 corresponds to normal sciatic nerve function, whereas –100 indicates complete functional impairment. At 12 weeks post-surgery, animals were euthanized, and the operated sciatic nerves were harvested for histological evaluation. Nerve samples were fixed in 4% paraformaldehyde, embedded in paraffin, and longitudinally sectioned at 5 µm thickness. Hematoxylin and eosin (H&E) staining was performed to assess general tissue morphology, cellular density, and fiber alignment. Luxol fast blue (LFB) staining was employed to evaluate myelin integrity and axonal regeneration, and Masson’s trichrome staining was used to examine connective tissue deposition. Histological images were acquired under a light microscope at ×200 magnification, and representative regions were analyzed for axonal organization, myelin thickness, inflammatory infiltration, and fibrosis [30].

### 2.10. Statistical Analysis

Statistical analyses were performed using Microsoft Excel. In vitro data are presented as mean ± SD (*n* = 3 independent replicates). Two-way ANOVA with Tukey’s post hoc test was used for swelling and degradation experiments, while unpaired *t*-tests were applied for cytotoxicity and suture retention tests. A *p* value < 0.05 was considered statistically significant. Pilot in vivo data are presented descriptively as mean ± SD. Due to the preliminary nature of the study and the limited sample size (*n* = 3 rats: SCT *n* = 1, CK154 *n* = 2), no statistical comparisons were performed and the results are reported qualitatively.

## 3. Results

### 3.1. Surface Chemistry Characterization

**Figure 2:**
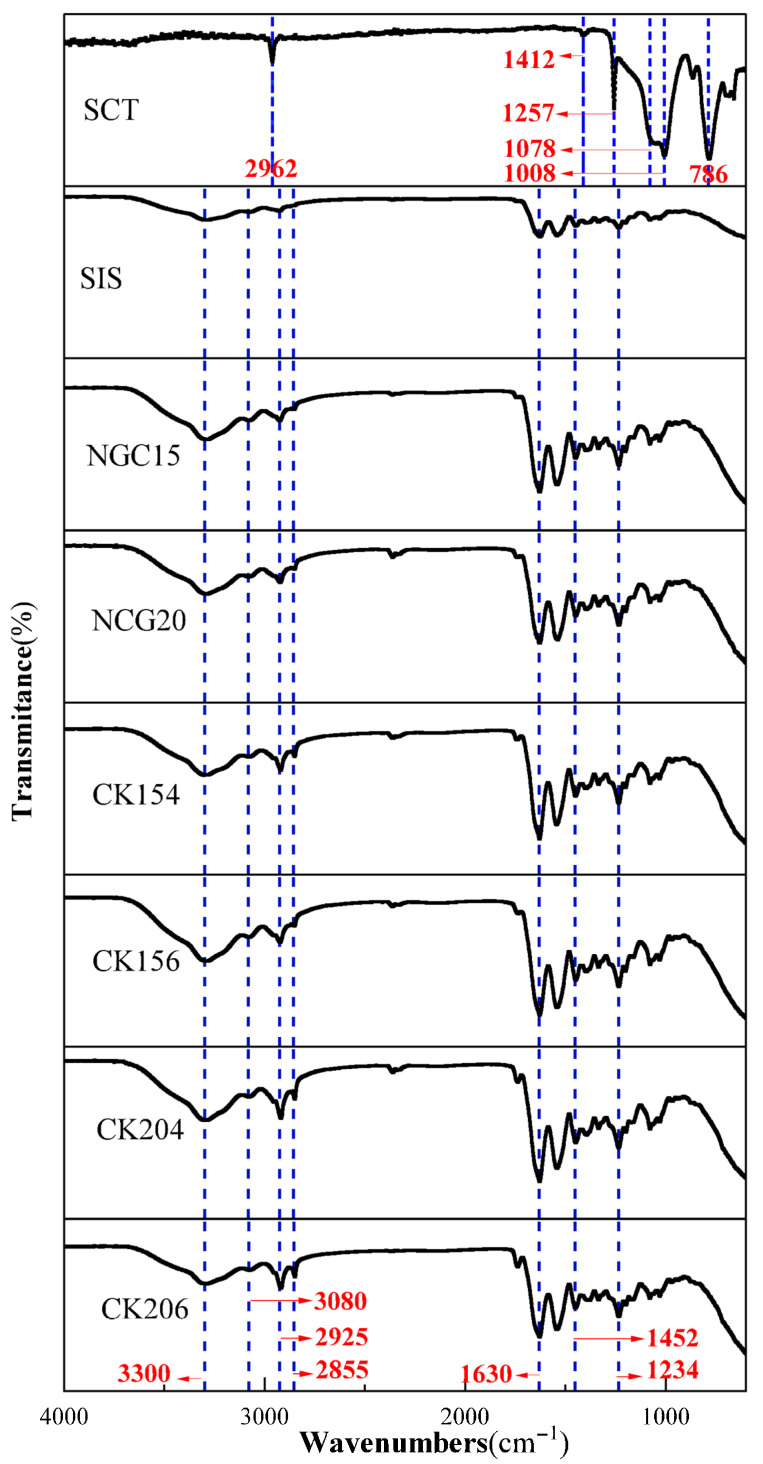
Fourier-transform infrared spectroscopy (FTIR) analysis of the braided SIS conduits. Comparison among SCT, SIS, NGCs, and braided conduits (CK154, CK156, CK204, CK206) showed that the braiding process did not alter the chemical composition of SIS, demonstrating that the bioactive components were preserved during conduit fabrication.

The FTIR spectra of the silicone conduit and the collagen-based conduit revealed distinct characteristic absorption bands corresponding to their chemical compositions. For the silicone conduit, absorption peaks were observed at 2962 cm^−1^, assigned to C–H stretching vibrations, 1412 cm^−1^ corresponding to CH_2_ bending, 1257 cm^−1^ attributed to Si–CH_3_ bending, 1078 and 1008 cm^−1^ associated with Si–O–Si stretching vibrations, and 786 cm^−1^ corresponding to Si–C stretching. These peaks are consistent with the typical spectrum of polydimethylsiloxane (PDMS)-based silicone materials (Figure 2).

The collagen conduit exhibited characteristic protein-related absorption bands. A broad band at 3300 cm^−1^ corresponds to N–H stretching vibrations (Amide A), while peaks at 3080 cm^−1^ are associated with =C–H stretching. The peaks at 2925 and 2855 cm^−1^ represent CH_2_ asymmetric and symmetric stretching vibrations (Amide B). A strong band at 1630 cm^−1^ (Amide I) arises from C=O stretching of peptide bonds, and the band at 1452 cm^−1^ corresponds to CH_2_ bending vibrations. Finally, the absorption at 1234 cm^−1^ (Amide III) is attributed to C–N stretching and N–H deformation. These spectral features are consistent with previously reported collagen data, confirming the preserved molecular structure of the collagen-based scaffold.

### 3.2. Decellularization and Surface Morphology of Nerve Guidance Conduits

**Figure 3:**
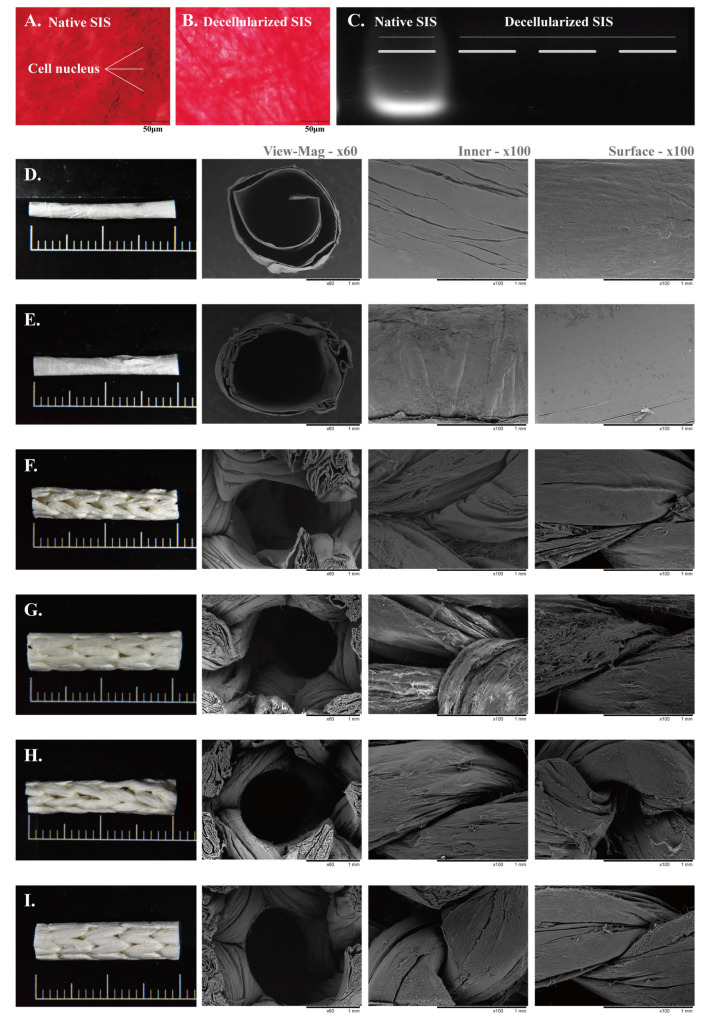
SIS decellularization verification and conduit morphology. (**A**) Native SIS showing abundant cell nuclei (EVG stain). (**B**) Decellularized SIS demonstrating the absence of cellular components. (**C**) Gel electrophoresis showing DNA bands in native SIS and their absence after decellularization, confirming effective DNA removal. (**D**–**I**) Representative conduit photographs and SEM images (gross view, cross-section, lumen, and outer surface) of rolled and braided NGCs.

The effectiveness of the SIS decellularization process was evaluated using histological staining and DNA analysis (Figure 3A–C). EVG staining of native SIS revealed abundant cellular components, as indicated by the presence of nuclei (Figure 3A). In contrast, no visible nuclei were observed in decellularized SIS (Figure 3B), suggesting effective removal of cellular content. This observation was further supported by gel electrophoresis, where distinct DNA bands were detected in native SIS, whereas no detectable bands were observed in decellularized samples (Figure 3C), indicating substantial removal of residual DNA.

The surface morphology of the nerve guidance conduits (NGCs) was examined using stereomicroscopy and scanning electron microscopy (SEM), as shown in Figure 3D–I. NGC15 (Figure 3D) and NGC20 (Figure 3E) exhibited conventional rolled tubular structures with smooth and compact walls, whereas the CK conduits (Figure 3F–I) displayed distinct braided architectures formed from SIS strips of different widths and braiding configurations. Cross-sectional SEM images revealed that the rolled conduits had dense and continuous wall structures, while the braided conduits exhibited interconnected fibrous networks with more open architectures.

SEM images of the luminal surface (100×, third column) showed smooth and uniform inner surfaces in the rolled conduits, whereas the braided conduits exhibited interwoven fiber structures, with increasing SIS strip width and braiding needle number resulting in more compact luminal architectures. Similarly, SEM images of the outer surface (100×, fourth column) showed smooth surfaces in the rolled conduits and interwoven fibrous morphologies in the braided conduits, with higher braiding density producing more tightly packed outer surfaces.

These results indicate that the braided design alters conduit microarchitecture and results in a more organized fibrous architecture compared with conventional rolled conduits. Importantly, the braided conduits maintained continuous tubular morphology, lumen patency, and structural integrity without observable collapse or delamination under SEM observation, suggesting their suitability for subsequent handling and implantation.

### 3.3. Swelling and Degradation Behavior

**Figure 4:**
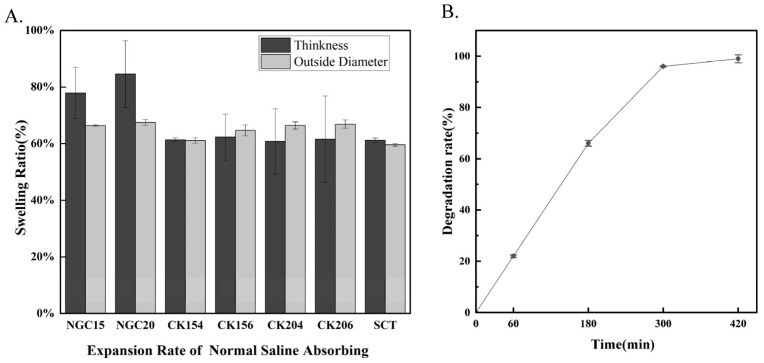
(**A**) Dimensional changes in the conduits after 1 h in physiological saline. All samples swelled in thickness, diameter, and length, with CK154 showing the highest dimensional stability. (**B**) Enzymatic degradation of CK154 braided SIS conduit only in Proteinase K. The conduits exhibited rapid mass loss, demonstrating good biodegradability of SIS-derived scaffolds under enzymatic conditions. Data are presented as mean ± SD (*n* = 3 independent replicates).

The swelling behavior of the conduits was assessed by measuring changes in thickness, outer diameter, and length (Figure 4A). The NGC group exhibited the highest percentage swelling, particularly in outer diameter, indicating greater water absorption and a less compact structure. Among the CK conduits, CK154 showed the lowest swelling ratios, suggesting superior dimensional stability, whereas CK156, CK204, and CK206 exhibited higher swelling due to looser braided architectures. The SCT silicone tube remained essentially unchanged, confirming its excellent size stability. Enzymatic degradation in Proteinase K demonstrated progressive mass loss over time (Figure 4B), with over 50% degradation within 180–240 min and nearly complete degradation (>95%) by 360–420 min. These results indicate that SIS-derived conduits are biodegradable, with a relatively rapid enzymatic degradation profile. Notably, CK154 exhibited lower swelling ratios compared with other braided designs, suggesting improved dimensional stability during hydration. This may contribute to maintaining structural integrity during early-stage application, while still allowing gradual degradation over time.

### 3.4. In Vitro Cytotoxicity of Nerve Guidance Conduits

**Figure 5:**
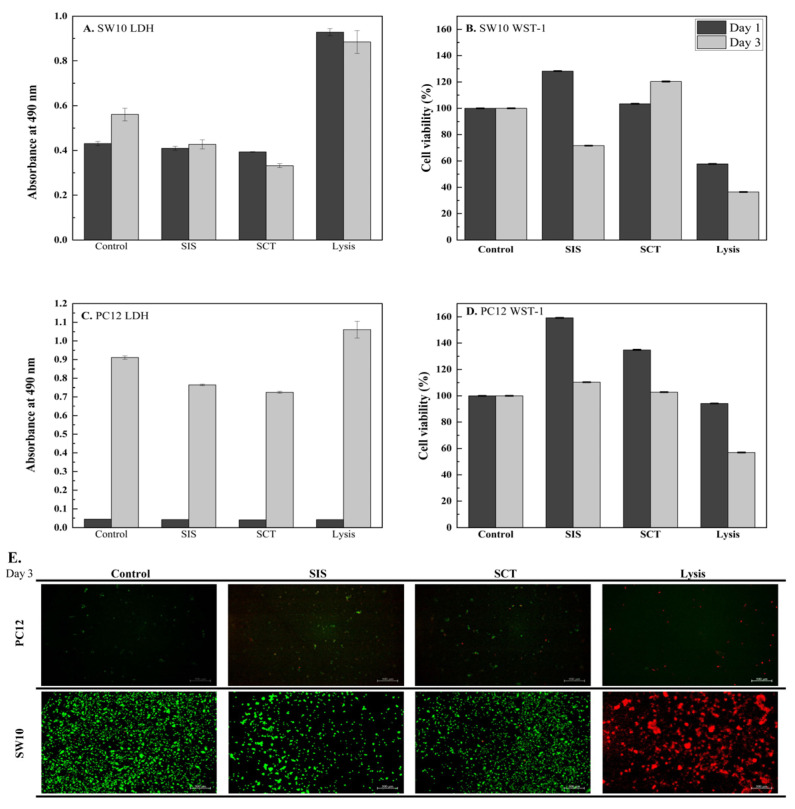
Cytotoxicity and biocompatibility evaluation of the conduits. (**A**,**B**) Lactate dehydrogenase (LDH) release and Cell Viability of SW10 cells cultured with conduit extraction medium. (**C**,**D**) LDH release and Cell Viability of PC12 cells cultured with conduit extraction medium. (**E**) Representative Live/Dead fluorescence images of SW10 and PC12 cells stained with calcein AM (live, green) and ethidium homodimer-1 (dead, red) after exposure to conduit extracts. Data are presented as mean ± SD (*n* = 3 independent replicates).

Cytotoxicity of SIS extracts was evaluated in SW10 (mouse Schwann cells) and PC12 (rat pheochromocytoma cells) using LDH release (Figure 5A,C) and WST-1 metabolic activity (Figure 5B,D) on Days 1 and 3, with Lysis and medium as positive and negative controls, respectively (*n* = 3, mean ± SD). In SW10 cells, LDH values remained low and comparable between the Control group (Day 1: 0.4307, Day 3: 0.5604) and the SIS group (Day 1: 0.4096, Day 3: 0.4273), while Lysis produced high release (0.9286). Correspondingly, Cell Viability of SW10 in the SIS group reached 128% of Control at Day 1 and 72% at Day 3, compared with 58% and 36% for Lysis. For PC12 cells, LDH values were minimal at Day 1 (Control: 0.0444, SIS: 0.0419) and increased by Day 3 (Control: 0.9110, SIS: 0.7637). Cell Viability of PC12 in the SIS group was 159% of Control at Day 1 and 110% at Day 3, compared with 94% and 57% for Lysis.

Live/Dead staining (Figure 5E, Day 3) showed predominantly viable cells in both cell types. Quantitative fluorescence analysis indicated viability above 96% for SW10 and approximately 85–87% for PC12 in SIS extracts. These results suggest that SIS exhibits favorable cytocompatibility, supporting higher cell viability and lower cytotoxicity compared with SCT [31].

### 3.5. Suture Retention and Tensile Properties of Conduits

**Figure 6:**
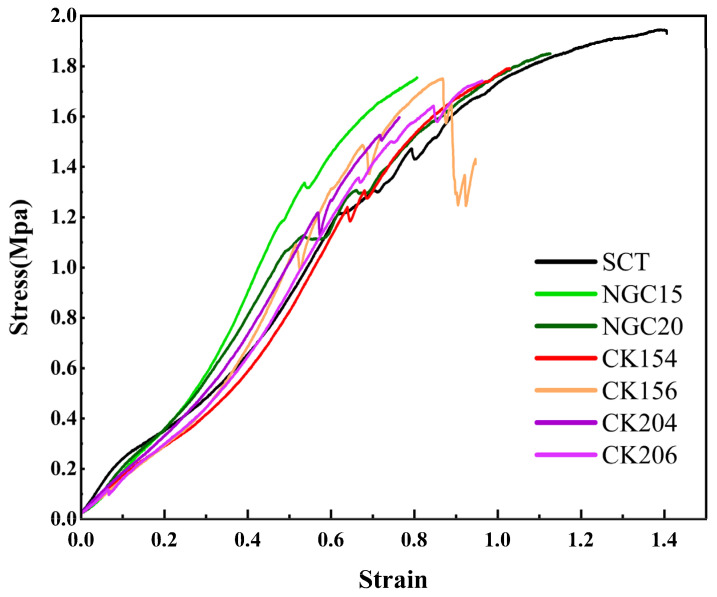
Nerve guidance conduit suture retention strength for operative usability. SCT showed the highest strength, followed by rolled SIS (NGC15, NGC20) and braided CK conduits, with CK154 exhibiting the highest strength among CK series. Data are presented as mean ± SD (*n* = 3 independent replicates).

The Suture retention and tensile properties of the conduits were evaluated using uniaxial tensile testing with 8/0 sutures secured at both ends of each sample (Table 1 and Figure 6). All conduits exhibited non-linear stress–strain behavior, characteristic of compliant soft biomaterials. The silicone tube (SCT) showed a smooth and continuous stress–strain profile with high extensibility, reaching approximately 2.0 MPa at maximum strain. Among the SIS conduits, the rolled NGC15 and NGC20 groups exhibited high ultimate tensile strength (approximately 1.80–1.85 MPa) and steeper elastic slopes, indicating greater stiffness compared with braided conduits. NGC20 demonstrated tensile strength approaching that of SCT, suggesting that the rolled structure provides effective mechanical reinforcement.

Within the braided CK series, mechanical properties varied with braiding configuration and material width. CK154 exhibited the highest tensile strength among braided conduits (1.793 MPa), followed by CK156 (1.742 MPa) and CK204 (1.597 MPa). CK154 also showed a stable stress–strain profile with suitable extensibility, whereas CK156 exhibited less uniform mechanical behavior, and CK204 and CK206 demonstrated lower strain capacity. CK154 provided the most favorable balance of tensile strength and extensibility among the degradable braided conduits. Its mechanical performance was lower than that of rolled SIS conduits but remained sufficient for suture fixation. Macroscopic and SEM observations confirmed that all conduits maintained tubular morphology, lumen patency, and braided or rolled architecture under mechanical testing, with no collapse, delamination, or fiber breakage, indicating preserved structural integrity. Based on these results, CK154 was selected for subsequent in vivo evaluation.

### 3.6. Bending Flexibility and Kink Resistance

**Figure 7:**
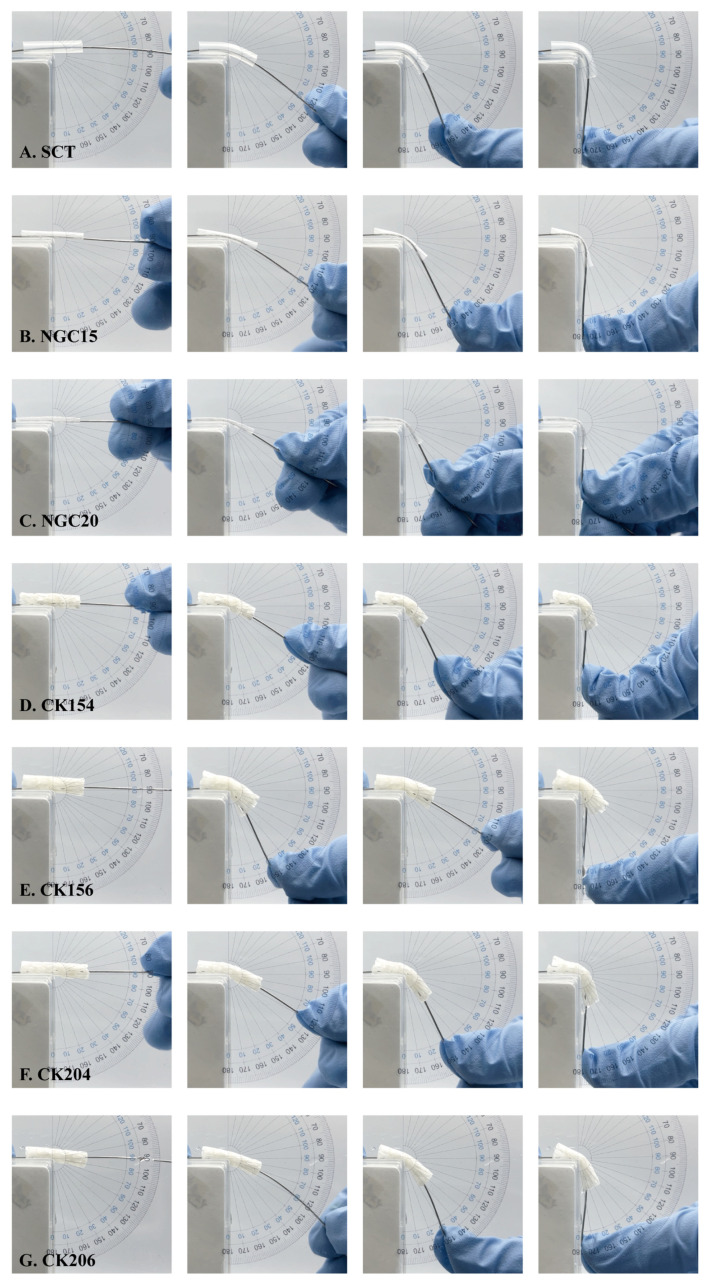
Bending and kink resistance of seven conduit groups. CK154 exhibited optimal flexibility, maintaining tubular structure and luminal patency without kinking up to 90°, outperforming CK156/204/206 while matching SCT integrity.

The bending and kink resistance of silicone conduits (SCT) and braided CK154 conduits were evaluated at bending angles of 0°, 30°, 60°, and 90° to assess flexibility and structural integrity (Figure 7). As shown in Figure 7A,F, both SCT and CK154 maintained their tubular structure without evident lumen collapse or kinking across all bending angles. The luminal architecture remained intact even at 90°, indicating good structural stability under mechanical deformation. CK154 exhibited bending behavior comparable to SCT, preserving luminal patency without visible fracture or structural failure. Observations during bending tests confirmed that the braided architecture of CK154 remained intact, with no visible delamination, fiber breakage, or lumen collapse, supporting the maintenance of tissue integrity under mechanical stress. These findings indicate that the braided CK154 conduit possesses sufficient flexibility and kink resistance to maintain structural integrity under bending conditions.

### 3.7. In Vivo Evaluation in a Rat Sciatic Nerve Transection Model

**Figure 8:**
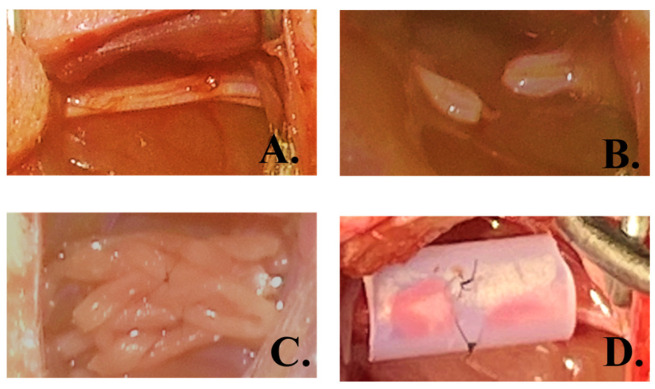
Rat sciatic nerve transection and conduit implantation. (**A**) Intact nerve. (**B**) Post-transection. (**C**) CK154 (8 mm) bridging gap. (**D**) SCT (8 mm) bridging gap. Both secured with 8-0 sutures.

A pilot in vivo study was conducted using Sprague-Dawley rats, with one animal in the silicone conduit (SCT) group and two animals in the CK154 conduit group. The surgical procedure is illustrated in Figure 8A–D, showing sciatic nerve exposure, transection to create a 10 mm defect, and conduit implantation secured with microsutures. Animals were monitored for 12 weeks post-implantation.

**Figure 9:**
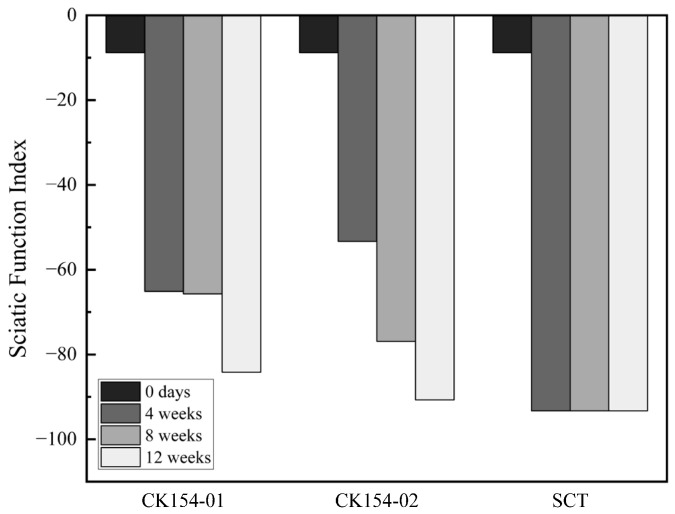
Sciatic Functional Index (SFI) over 12 weeks post-surgery. Silicone (SCT) conduits showed persistently low SFI due to autotomy and digit loss, whereas CK154 braided conduits exhibited minimal SFI changes due to toe flexion, indicating partial functional recovery despite morphological limitations.

**Figure 10:**
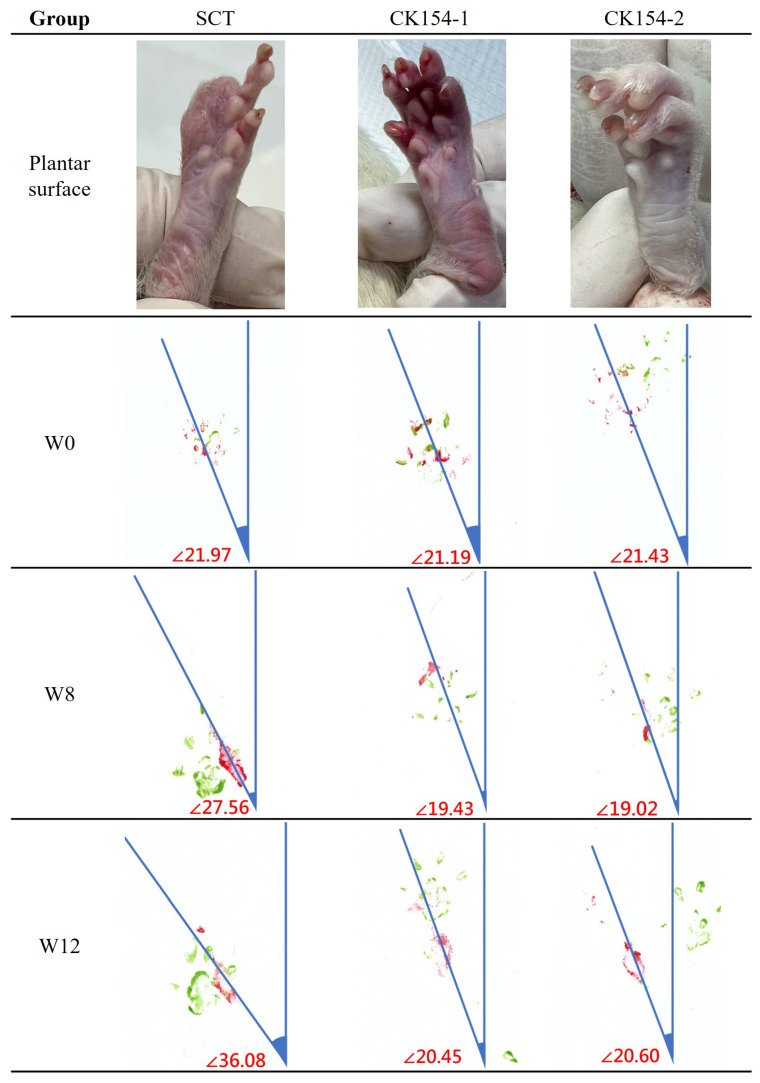
Toe-out angle analysis (green: left front paw; red: left rear paw). SCT displayed progressive gait deviations from autotomy, while CK154 maintained stable paw alignment despite toe deformities.

Functional recovery was evaluated using the Sciatic Functional Index (SFI) and toe-out angle analysis over 12 weeks post-surgery (Figure 9 and Figure 10). In the silicone conduit (SCT) group, severe autotomy occurred by week 4, resulting in digit loss, persistently low SFI values (~−100), and progressively increased toe-out angles (27.56° at week 8; 36.08° at week 12), indicating abnormal gait and profound functional deficits. These changes highlight the limitations of nondegradable conduits in supporting regeneration and the reduced reliability of footprint-based measurements when paw integrity is compromised.

In contrast, the CK154 braided conduit group showed no autotomy, demonstrating good tolerability. Persistent toe flexion limited full paw extension, resulting in minimal changes in SFI. However, toe-out angle analysis revealed only mild deviations from baseline (19.02° at week 8; 20.60° at week 12), suggesting partial restoration of coordinated paw placement and preserved limb alignment. Subtle improvements in limb positioning and locomotor patterns indicate ongoing axonal regeneration and partial motor recovery despite morphological limitations.

### 3.8. Gross and Histological Evaluation

**Figure 11:**
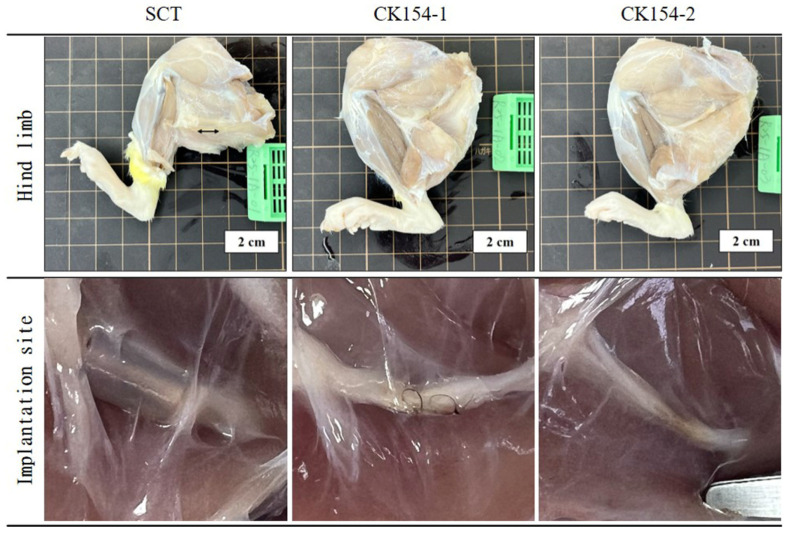
Gross evaluation of explanted rat hind limbs at 12 weeks post-implantation. Regenerated sciatic nerves bridged the 10 mm defect in both groups. Silicone (SCT) conduits remained intact, whereas CK154 conduits were fully degraded, with only residual sutures visible, indicating complete in vivo resorption.

**Figure 12:**
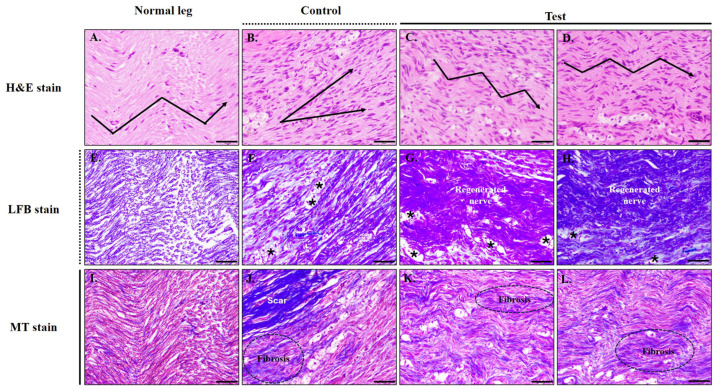
Histological evaluation of regenerated sciatic nerves at 12 weeks post-implantation (scale bar = 50 µm; arrows: direction of axon dispersion/growth). Longitudinal sections were stained with H&E (**A**–**D**), Luxol Fast Blue (LFB, **E**–**H**), and Masson’s Trichrome (MT, **I**–**L**). Normal nerves (**A**,**E**,**I**) exhibited well-aligned axons, uniform myelin, and minimal connective tissue. The silicone conduit (SCT) group (**B**,**F**,**J**) showed disorganized axons, myelin degeneration (*), increased cellularity, and extensive fibrosis. In contrast, the CK154 group (**C**–**D**, **G**–**H**, **K**–**L**) exhibited more organized axonal alignment, more continuous myelin sheaths, and reduced fibrotic tissue compared with the SCT group. Descriptive data are presented as mean ± SD (*n* = 3 rats: SCT *n* = 1, CK154 *n* = 2). No statistical analysis was performed due to pilot study design.

At 12 weeks post-implantation, operated hind limbs were harvested for gross and histological assessment to evaluate conduit integrity and nerve regeneration (Figure 11 and Figure 12). Gross inspection revealed that silicone conduits (SCT) remained structurally intact, consistent with their nondegradable synthetic nature. In contrast, no intact CK154 conduits were observed; only residual sutures were detectable at the implantation site, indicating complete in vivo degradation of the collagen-based braided conduit. These findings demonstrate the intrinsic biodegradability of CK154, which allows gradual resorption without compromising initial mechanical guidance for early axonal growth. Complete degradation also eliminates the need for secondary removal procedures and reduces the risk of long-term foreign-body reactions. Despite differences in material persistence, regenerated sciatic nerves successfully bridged the 10 mm defect in both groups, indicating that CK154 provides sufficient early structural support while permitting natural tissue remodeling and maintenance of tissue integrity (Figure 11).

Histological analysis using H&E, Luxol Fast Blue (LFB), and Masson’s Trichrome (MT) staining revealed distinct differences among groups at 12 weeks post-implantation (Figure 12). Normal sciatic nerves exhibited characteristic wavy axonal alignment, low cellular density, and intact myelin sheaths (Figure 12A,E,I). In contrast, the SCT group showed disorganized axonal structures, increased cellularity, vacuolated and degenerated myelin fibers (*), and extensive fibrotic tissue infiltration (Figure 12B,F,J), suggesting incomplete nerve regeneration.

By comparison, the CK154 group demonstrated more organized axonal alignment, thicker and more continuous myelin sheaths, and reduced connective tissue deposition (Figure 12C,D,G,H,K,L), indicating preserved tissue integrity. Although mild to moderate fibrosis was occasionally observed, the overall architecture of regenerated nerves more closely resembled that of normal sciatic nerves. LFB and MT staining further confirmed improved myelin integrity and reduced fibrosis in the CK154 group compared with the SCT group. These observations collectively support that CK154 conduits maintain tissue structural integrity while promoting morphological recovery following peripheral nerve injury.

## 4. Discussion

The present study provides a comprehensive evaluation of CK154, a biodegradable collagen-based braided conduit, for peripheral nerve regeneration [32]. The in vitro and in vivo results collectively demonstrate that CK154 integrates mechanical resilience, gradual biodegradability, and a biologically permissive microenvironment to support organized axonal regeneration and partial functional recovery. Braided SIS nerve conduits preserved essential collagen structures and exhibited favorable mechanical, swelling, and enzymatic-degradation properties, which are critical for maintaining structural and tissue integrity while permitting progressive tissue remodeling [33,34,35]. The interconnected porous architecture likely facilitates nutrient diffusion, Schwann cell migration, and axonal elongation, all fundamental processes for effective nerve repair [36]. Importantly, the braided design conferred mechanical flexibility sufficient to preserve luminal and structural integrity during limb movement, consistent with established criteria for nerve guidance conduits [37].

Mechanically, CK154 exhibited a balanced combination of tensile strength and extensibility, sufficient to withstand suture fixation and physiological deformation [38]. While non-degradable silicone exhibited higher ultimate tensile strength and uniform stress–strain behavior, these synthetic constructs remain inert in vivo and do not support progressive tissue remodeling. The braided architecture of CK154 enabled uniform stress distribution along the conduit wall, maintained luminal patency under bending and kink stress, and supported overall tissue integrity, providing reliable guidance for regenerating axons. Such mechanical features are essential during early-stage regeneration, as they prevent structural collapse while allowing progressive load transfer to regenerating tissue, thereby promoting maturation of newly formed nerve fibers [39].

Biodegradability represents a major advantage of CK154. Gross examination at 12 weeks post-implantation confirmed complete resorption of the conduit, with only residual suture material detectable at the implantation site (Figure 12). Gradual degradation permits tissue replacement without compromising early mechanical guidance, while minimizing the risk of chronic foreign-body reactions and obviating the need for secondary removal procedures. Despite complete resorption, regenerated sciatic nerves maintained structural integrity and successfully bridged the 10 mm defect in all cases, indicating that CK154 provides adequate early support while enabling natural tissue remodeling.

Histological analyses further demonstrated the regenerative benefits of CK154. H&E, Luxol Fast Blue, and Masson’s Trichrome staining revealed that SCT-treated nerves exhibited disorganized axonal alignment, vacuolated myelin fibers, and extensive fibrosis, indicative of prolonged inflammatory responses and limited tissue integration (Figure 12). Dense scar tissue adhering to regenerated fibers suggested that persistent synthetic conduits may restrict microenvironmental permissiveness, potentially limiting functional recovery. In contrast, CK154-treated nerves displayed orderly axonal alignment, thicker and more continuous myelin sheaths, and substantially reduced connective tissue deposition, collectively reflecting preserved tissue integrity. Occasional moderate fibrosis likely reflects normal remodeling, but overall tissue architecture closely resembled that of uninjured nerves. These findings emphasize the importance of scaffold composition and architecture in modulating the local regenerative environment. The observed histological improvements are attributable to multiple features inherent to CK154: the collagen-based composition provides natural biochemical cues that promote cell adhesion and axonal elongation; the braided architecture creates interconnected porosity that enhances nutrient diffusion, vascular infiltration, and Schwann cell migration; and the gradual degradation allows progressive transfer of mechanical load to regenerating tissue, thereby supporting both structural and tissue integrity during functional remodeling [40]. Collectively, these properties reconcile the need for early mechanical stability with the biological requirement for scaffold resorption, a balance not typically achieved with non-degradable synthetic conduits.

Functionally, CK154 conduits were associated with improved gait parameters and maintenance of limb alignment, as evidenced by toe-out angle measurements [41]. Although Sciatic Functional Index (SFI) scores were constrained by persistent toe flexion deformities, the preservation of coordinated paw placement and absence of autotomy indicate ongoing axonal reconnection and partial motor recovery. These observations, together with preserved conduit and tissue integrity, highlight that effective peripheral nerve repair requires not only structural guidance but also a biologically permissive environment to support axonal regrowth and functional reinnervation.

By combining tailored mechanical properties, controlled biodegradation, and favorable histological outcomes, CK154 addresses limitations commonly associated with persistent synthetic conduits, including chronic foreign-body reactions, scar formation, and restricted tissue integration. Moreover, the braided architecture allows tunable mechanical and structural properties, providing a flexible platform for future optimization of conduit width, braiding density, and degradation kinetics to accommodate specific injury contexts.

In experimental peripheral nerve regeneration studies, silicone tubes have frequently been used as non-degradable inert conduits that provide a stable tubular structure for evaluating the regenerative environment within the conduit. Although silicone conduits are not clinically preferred materials, they serve as useful reference controls for assessing the biological performance of newly developed biomaterial conduits. The absence of an autograft control group represents a limitation of the present pilot study and will be considered in future investigations. A limitation of the present study is the relatively small sample size and the absence of quantitative morphometric analysis. Future studies with larger cohorts will include detailed measurements, such as axon density, myelin thickness, and g-ratio, to provide a more comprehensive evaluation of nerve regeneration and to further validate the regenerative performance of the SIS conduit.

In this pilot feasibility study (*n* = 3 total), preliminary indications of functional recovery (SFI) and muscle preservation were observed in the SIS braided conduit group (Figure 10 and Figure 11), despite the presence of foreign body response. These observations support the surgical feasibility and basic performance of the braided SIS design. This animal experiment was designed as a proof-of-concept pilot study to evaluate surgical handling, degradation behavior, and preliminary functional outcomes of the braided SIS conduit. Although encouraging observations were obtained, the small sample size precludes statistical comparisons. Larger cohort studies with additional control groups will be required to confirm these preliminary findings.

In summary, CK154 demonstrates considerable promise as a degradable scaffold for peripheral nerve repair. Its ability to provide early mechanical support while preserving tissue integrity, promote organized axonal regeneration, reduce fibrosis, and facilitate functional recovery distinguishes it from non-degradable alternatives. These findings provide a foundation for further preclinical investigations involving larger cohorts and extended follow-up to fully elucidate the relationship between scaffold degradation, tissue remodeling, and long-term functional outcomes. Future studies may also explore the incorporation of bioactive molecules or cell-based therapies within the braided conduit to further enhance regenerative potential, underscoring the versatility and translational relevance of CK154 in nerve tissue engineering [42].

## 5. Conclusions

Biodegradable braided SIS (CK154) nerve conduits exhibited a favorable combination of mechanical stability, dimensional integrity, biocompatibility, and controlled enzymatic degradability, supporting their potential use as scaffolds for peripheral nerve regeneration. In vivo, CK154 conduits facilitated organized axonal regrowth, preserved myelin structure, and minimized fibrotic and inflammatory responses. Functional evaluations suggested indicated that CK154 conduits supported limb alignment and gait coordination, despite minor toe deformities, indicating their capacity to provide early structural guidance and a permissive microenvironment for neural recovery. Although these findings are preliminary and derived from a small pilot study, they highlight the promise of biodegradable, collagen-based braided conduits as a strategy for peripheral nerve repair, providing both structural support and a biologically favorable microenvironment conducive to nerve tissue remodeling.

## Figures and Tables

**Table 1:** Mechanical properties and suture retention strength of nerve guidance conduits.

Sample	Suture Retention (N)	UTS (MPa)	Young’s Modulus (MPa)
SCT	1.40 ± 0.06	1.16 ± 0.22	1.79 ± 0.28
NGC15	1.29 ± 0.04	0.82 ± 0.12	2.31 ± 0.38
NGC20	1.26 ± 0.23	0.97 ± 0.18	1.81 ± 0.18
CK154	1.20 ± 0.15	1.13 ± 0.36	1.60 ± 0.48
CK156	1.29 ± 0.04	0.99 ± 0.05	1.86 ± 0.12
CK204	1.09 ± 0.20	0.80 ± 0.12	1.91 ± 0.63
CK206	1.30 ± 0.05	0.94 ± 0.02	2.13 ± 0.22

Values are presented as mean ± SD (*n* = 3). Statistical analysis was performed using one-way ANOVA, and no statistically significant differences were observed among groups (*p* > 0.05). Suture retention strength is expressed in N, and mechanical properties are expressed in MPa.

## Data Availability

The original contributions presented in the study are included in the article, further inquiries can be directed to the corresponding author.

## Abbreviations

The following abbreviations are used in this manuscript:

bFGF	basic Fibroblast Growth Factor
CNS	Central Nervous System
DHT	Dehydrothermal
ECM	Extracellular Matrix
FTIR	Fourier-Transform Infrared Spectroscopy
GAGs	Glycosaminoglycans
NGC	Nerve Guidance Conduit
PNI	Peripheral Nerve Injury
PNS	Peripheral Nervous System
SEM	Scanning Electron Microscopy
SIS	Small Intestinal Submucosa

## References

[1] Li X., Zhang X., Hao M., Wang D., Jiang Z., Sun L., Gao Y., Jin Y., Lei P., Zhuo Y. (2022). The application of collagen in the repair of peripheral nerve defect. Front. Bioeng. Biotechnol..

[2] Asplund M., Nilsson M., Jacobsson A., von Holst H. (2009). Incidence of traumatic peripheral nerve injuries and amputations in Sweden between 1998 and 2006. Neuroepidemiology.

[3] Feigin V.L., Vos T., Nichols E., Owolabi M.O., Carroll W.M., Dichgans M., Deuschl G., Parmar P., Brainin M., Murray C. (2020). The global burden of neurological disorders: Translating evidence into policy. Lancet Neurol..

[4] Yi S., Zhang Y., Gu X., Huang L., Zhang K., Qian T., Gu X. (2020). Application of stem cells in peripheral nerve regeneration. Burn. Trauma.

[5] Manoukian O.S., Baker J.T., Rudraiah S., Arul M.R., Vella A.T., Domb A.J., Kumbar S.G. (2020). Functional polymeric nerve guidance conduits and drug delivery strategies for peripheral nerve repair and regeneration. J. Control. Release.

[6] Kong Y., Xu J., Guan W., Sun S., Yang Y., Li G. (2023). Tailoring the elasticity of nerve implants for regulating peripheral nerve regeneration. Smart Mater. Med..

[7] Daly W., Yao L., Zeugolis D., Windebank A., Pandit A. (2012). A biomaterials approach to peripheral nerve regeneration: Bridging the peripheral nerve gap and enhancing functional recovery. J. R. Soc. Interface.

[8] Gao Y., Wang Y.L., Kong D., Qu B., Su X.J., Li H., Pi H.Y. (2015). Nerve autografts and tissue-engineered materials for the repair of peripheral nerve injuries: A 5-year bibliometric analysis. Neural Regen. Res..

[9] Modo M., Lampe K. (2019). Development and implementation of biomaterials to promote neural repair. Brain Res. Bull..

[10] Ducic I., Fu R., Iorio M.L. (2012). Innovative treatment of peripheral nerve injuries: Combined reconstructive concepts. Ann. Plast. Surg..

[11] Zhang X., Yao L., Yan Y., Fu M. (2024). Evolution of natural polymer nerve conduit technology in peripheral nerve repair: A narrative review. Adv. Technol. Neurosci..

[12] Costa M.P., Teixeira N.H., Longo M.V., Gemperli R., Costa H.J. (2015). Combined polyglycolic acid tube and autografting versus autografting or polyglycolic acid tube alone. A comparative study of peripheral nerve regeneration in rats. Acta Cir. Bras..

[13] Sarker M., Naghieh S., McInnes A.D., Schreyer D.J., Chen X. (2018). Strategic Design and Fabrication of Nerve Guidance Conduits for Peripheral Nerve Regeneration. Biotechnol. J..

[14] Zhang S., Wang J., Zheng Z., Yan J., Zhang L., Li Y., Zhang J., Li G., Wang X., Kaplan D. (2021). Porous nerve guidance conduits reinforced with braided composite structures of silk/magnesium filaments for peripheral nerve repair. Acta Biomater..

[15] Houshyar S., Bhattacharyya A., Shanks R. (2019). Peripheral Nerve Conduit: Materials and Structures. ACS Chem. Neurosci..

[16] Arslantunali D., Dursun T., Yucel D., Hasirci N., Hasirci V. (2014). Peripheral nerve conduits: Technology update. Med. Devices Evid. Res..

[17] Li J., Ren N., Qiu J., Jiang H., Zhao H., Wang G., Boughton R.I., Wang Y., Liu H. (2013). Carbodiimide crosslinked collagen from porcine dermal matrix for high-strength tissue engineering scaffold. Int. J. Biol. Macromol..

[18] Fujii M., Tanaka R. (2022). Porcine Small Intestinal Submucosa Alters the Biochemical Properties of Wound Healing: A Narrative Review. Biomedicines.

[19] Zhao Y., Peng H., Sun L., Tong J., Cui C., Bai Z., Yan J., Qin D., Liu Y., Wang J. (2024). The application of small intestinal submucosa in tissue regeneration. Mater. Today Bio.

[20] Rummler L.S., Gupta R. (2004). Peripheral nerve repair: A review. Curr. Opin. Orthop..

[21] Du J., Chen H., Qing L., Yang X., Jia X. (2018). Biomimetic neural scaffolds: A crucial step towards optimal peripheral nerve regeneration. Biomater. Sci..

[22] Batasheva S., Kotova S., Frolova A., Fakhrullin R. (2024). Atomic force microscopy for characterization of decellularized extracellular matrix (dECM) based materials. Sci. Technol. Adv. Mater..

[23] Li Y.X., Zhao L.M., Zhang X.Z., Ma X.K., Liang J.Q., Gan T.J., Gong H., Jiang Y.L., Wu Y., Song Y.T. (2025). Smooth muscle extracellular matrix modified small intestinal submucosa conduits promote peripheral nerve repair. Biomaterials.

[24] Zhukauskas R., Fischer D.N., Deister C., Faleris J., Marquez-Vilendrer S.B., Mercer D. (2023). Histological Comparison of Porcine Small Intestine Submucosa and Bovine Type-I Collagen Conduit for Nerve Repair in a Rat Model. J. Hand Surg. Glob. Online.

[25] Lin K.-Y., Tsai Y.-L., Kuan C.-Y., Lin Y.-C., Su W.-Y., Chang H.-H., Wang C.-Y. (2026). A preclinical overall evaluation of a novel biomimetic collagen membrane for guided tissue regeneration. J. Dent. Sci..

[26] (2021). Biological Evaluation of Medical Devices—Part 12: Sample Preparation and Reference Materials.

[27] Babovic N., Klaus D., Schessler M.J., Schimoler P.J., Kharlamov A., Miller M.C., Tang P. (2019). Assessment of Conduit-Assisted Primary Nerve Repair Strength with Varying Suture Size, Number, and Location. Hand.

[28] Kim J., Park J., Choe G., Jeong S.I., Kim H.S., Lee J.Y. (2024). A Gelatin/Alginate Double Network Hydrogel Nerve Guidance Conduit Fabricated by a Chemical-Free Gamma Radiation for Peripheral Nerve Regeneration. Adv. Healthc. Mater..

[29] Varejao A.S., Meek M.F., Ferreira A.J., Patricio J.A., Cabrita A.M. (2001). Functional evaluation of peripheral nerve regeneration in the rat: Walking track analysis. J. Neurosci. Methods.

[30] Carriel V., Garzon I., Alaminos M., Cornelissen M. (2014). Histological assessment in peripheral nerve tissue engineering. Neural Regen. Res..

[31] Gallo N., Lunetti P., Bettini S., Barca A., Madaghiele M., Valli L., Capobianco L., Sannino A., Salvatore L. (2020). Assessment of physico-chemical and biological properties of sericin-collagen substrates for PNS regeneration. Int. J. Polym. Mater. Polym. Biomater..

[32] Nicholls K., Furness N.D. (2019). Peripheral nerve compression syndromes of the upper limb. Surgery.

[33] Agarwalla A., Ahmed W., Al-Marzouqi A.H., Zaneldin E., Rizvi T.A., Khan M. (2025). Advancements in synthetic polymers for 3D bioprinting materials, applications, and future prospects. Int. J. Polym. Mater. Polym. Biomater..

[34] Toth T., Prisca R.A., Ballo N., Prisca A.M., Szasz E.A., Borda A. (2025). Porcine Small Intestinal Submucosa Extracellular Matrix: A Meta-Analysis of Composition, Processing Techniques, and Biomedical Applications. Int. J. Mol. Sci..

[35] Alhosseini S.N., Moztarzadeh F., Kargozar S., Dodel M., Tahriri M. (2015). Development of Polyvinyl Alcohol Fibrous Biodegradable Scaffolds for Nerve Tissue Engineering Applications:In Vitro Study. Int. J. Polym. Mater. Polym. Biomater..

[36] Keane T.J., Swinehart I.T., Badylak S.F. (2015). Methods of tissue decellularization used for preparation of biologic scaffolds and in vivo relevance. Methods.

[37] Bejar-Chapa M., Rossi N., King N.C., Kostyra D.M., Hussey M.R., McGuire K.R., Randolph M.A., Redmond R.W., Winograd J.M. (2025). Comparison of Photochemically Sealed Commercial Biomembranes for Nerve Regeneration. J. Funct. Biomater..

[38] Monfette V., Choiniere W., Godbout-Lavoie C., Pelletier S., Langelier E., Lauzon M.A. (2023). Thermoelectric Freeze-Casting of Biopolymer Blends: Fabrication and Characterization of Large-Size Scaffolds for Nerve Tissue Engineering Applications. J. Funct. Biomater..

[39] Delgado L.M., Pandit A., Zeugolis D.I. (2014). Influence of sterilisation methods on collagen-based devices stability and properties. Expert Rev. Med. Devices.

[40] Shen Y., Xu Y., Chen J. (2024). Advanced biomaterials for tendon repair: Development and application. Int. J. Polym. Mater. Polym. Biomater..

[41] Lakes E.H., Allen K.D. (2016). Gait analysis methods for rodent models of arthritic disorders: Reviews and recommendations. Osteoarthr. Cartil..

[42] Li T., Javed R., Ao Q. (2021). Xenogeneic Decellularized Extracellular Matrix-based Biomaterials for Peripheral Nerve Repair and Regeneration. Curr. Neuropharmacol..

